# Notes and News

**Published:** 1892-02-06

**Authors:** 


### Notes and News.

Collected and Communicated.

The City of London Truss Society has done a good work during the past year. About 9,000 instruments have been given to necessitous patients. The great value of the work of such a society as this is that in very many cases it prevents patients becoming a burden to their friends or a charge upon the public, and in order that necessitous persons may be relieved promptly, the surgeons are in attendance every morning throughout the year (Sundays excepted).

The Tunbridge Wells and Rusthall Provident Dispensary is doing excellent work. The total receipts amount to £734, the largest sum received in any year except one. The expenditure amounted to £722, of which £410 represents payments made to the Medical Officers. The number of members on the books during the year has been 2,503, of whom 2,075 remained at the end of the year, 1,305 of them being above the age of fourteen. Members pay three half-pence per week if over, and one penny per week if under fourteen years of age. Having regard to the enormous number of out-patients attending our hospitals, these figures are instructive, and should excite the earnest consideration of hospital managers all over the country.

The Radcliffe Infirmary, Oxford, has to commence the year with a deficit of £470. Much of the speech of the Master of University, who presided at the quarterly meeting of the Governors on the 27th ult., might have been delivered by our Special Commissioner. Speaking of the appeal, he said, "What we have done is to try and call the county and city and University back to the view that they have a real duty to perform in maintaining what is a piece of their public property, and if we can move them to that there will be some chance, perhaps, of making this place what it ought to be—a very excellent and perfect hospital. But that matter lies in the hands, not of the Committee, nor even of the Governors, but of the whole county and city of Oxford. If they choose to have a second-rate place, carried on penuriously and from hand to mouth, they can have it. If they choose, on the other hand, to have a handsome, well-equipped hospital, doing its work thoroughly and well, as it ought to do, they can have it, but they must pay for it, and the matter, as far as I can see, is now left in their hands. If they refuse to help us, I think our effort will drop, and we shall then have to do the best we can to continue the work in a second-rate place." Precisely. This is all we have been contending for since we first called public attention to the urgent need there is for a thorough renovation and reconstruction of the Radcliffe Infirmary at Oxford. We congratulate the Treasurer on the courage he has displayed, and hope his words of wisdom may find an echo in the hearts of the people of the city and county of Oxford.

For some time past the *British Medical Journal* has been carefully looking into the case of the Irish dispensary doctors. The mass of evidence laid before us demonstrates the necessity of a champion for the cause. To make a long story short, we gather that £140 is a fair average of the total official income of an Irish dispensary doctor. His duty occasions him to traverse a large area as a rule—private practices are worth very little—and the doctor is practically a prisoner to his district, no holidays being permissible unless a substitute is paid by the doctor himself, and sick leave is in many cases hard to obtain. These are, of course, only the bare outlines of the case. The details appeal to our humanity and sense of justice. Some of the poor-law medical officers of County Waterford have held a meeting on the subject of their grievances, stimulated by the advocacy of the *British Medical Journal*, and it is intended, if possible, to lay the case before Parliament, that some reform may be effected. The grievance of the Irish poor-law doctors is a genuine one, and calls for effective and speedy removal.

The necessity which has arisen for a considerable increase in the ward accommodation of the Edinburgh Royal Infirmary, has called forth more than one plan of reconstruction. Although the world moves by differences of opinion it is desirable, with a view to secure the combined support of the promoters of each plan, to refer the whole to a representative committee, with power to take evidence and to report which is the most suitable in the best interests of Scotland. One proposal, which would provide accommodation for 200 extra patients, is estimated to cost £73,500. Dr. Clouston, however, urges that an Act of Parliament should be obtained, that further ground should be purchased, and that an expenditure of from £150,000 to £200,000 should be at once entered upon. The enormous difference between the outlay on the two schemes induced the Governors at their meeting, on Monday week, to wisely pause before coming to a final decision. The various questions at issue have, therefore, been referred to an adjourned meeting, to be held a month hence. It appears that the practice prevails of sending sick servants to the Infirmary, and that whenever the master or mistress finds the beds are full, he or she is very apt to withdraw or reduce the subscription. More than two hundred householders, the *Scotsman* declares, in the space of one month have refused to subscribe to the Infirmary. Controversy has arisen as to the adequate development of the Medical School, and the provision of facilities for the teaching of women. We shall await further developments with interest, and hope soon to be able to record a wise solution of the many difficulties referred to.

On the 21st inst. the National Surgical Institute, Indianapolis, U.S.A., was burnt down. It contained some hundreds of patients, men, women, and children, and as the fire occurred after midnight a panic ensued, which resulted in much lamentable loss of life, nineteen bodies having been recovered from the ruins. It would be interesting to know how many hospitals have a fire drill regularly every week in some portion of the building, so as to ensure that the whole of the fire extinguishing appliances are thoroughly tested at least once a month. Messrs. Merryweather and Sons have perfected a system of fire prevention in hospitals and large establishments which managers would be well advised to make themselves familiar with. Every English hospital which has not yet instituted a fire brigade drill should take steps to do so without delay. We remember in our experience a fire at a great hospital in the provinces, from which upwards of forty fever patients in a most dangerous condition were successfully removed, and comfortably placed in fresh beds in the short period of twenty minutes. This was done, although the fire was most severe and rapid in its course. A large number of these poor patients must have been burned to death had it not been for the excellent discipline which prevailed in the hospital in question, and the readiness with which every member of the staff fell into his or her place on the first alarm of fire. There is good reason to fear that many English institutions have no adequate system of protection against fire. Let us, therefore, take warning by the fire at Indianapolis, so that such a scene cannot possibly be repeated in any institution throughout the British Empire.

